# Statistical analyses of ordinal outcomes in randomised controlled trials: protocol for a scoping review

**DOI:** 10.1186/s13063-023-07262-8

**Published:** 2023-04-21

**Authors:** Chris J. Selman, Katherine J. Lee, Clare L. Whitehead, Brett J. Manley, Robert K. Mahar

**Affiliations:** 1 Clinical Epidemiology and Biostatistics Unit, Murdoch Children’s Research Institute, Parkville, VIC 3052 Australia; 2 Department of Paediatrics, University of Melbourne, Parkville, VIC 3052 Australia; 3 Department of Obstetrics and Gynaecology, University of Melbourne, Parkville, VIC 3052 Australia; 4 Department of Maternal Fetal Medicine, The Royal Women’s Hospital, Parkville, VIC 3052 Australia; 5 Newborn Research Centre, The Royal Women’s Hospital, Parkville, VIC 3052 Australia; 6 Neonatal Research, Murdoch Children’s Research Institute, Parkville, VIC 3052 Australia; 7 Centre for Epidemiology and Biostatistics, Melbourne School of Population and Global Health, University of Melbourne, Parkville, VIC 3052 Australia

**Keywords:** Ordinal outcomes, Scoping review, Randomised controlled trials

## Abstract

**Introduction:**

Randomised controlled trials (RCTs) aim to assess the effect of one (or more) unproven health interventions relative to other reference interventions. RCTs sometimes use an ordinal outcome, which is an endpoint that comprises of multiple, monotonically ordered categories that are not necessarily separated by a quantifiable distance. Ordinal outcomes are appealing in clinical settings as specific disease states can represent meaningful categories that may be of clinical importance to researchers. Ordinal outcomes can also retain information and increase statistical power compared to dichotomised outcomes and can allow multiple clinical outcomes to be comprised in a single endpoint. Target parameters for ordinal outcomes in RCTs may vary depending on the nature of the research question, the modelling assumptions and the expertise of the data analyst.

The aim of this scoping review is to systematically describe the use of ordinal outcomes in contemporary RCTs. Specifically, we aim to:

$\bullet$ Identify which target parameters are of interest in trials that use an ordinal outcome, and whether these parameters are explicitly defined.

$\bullet$ Describe how ordinal outcomes are analysed in RCTs to estimate a treatment effect.

$\bullet$ Describe whether RCTs that use an ordinal outcome adequately report key methodological aspects specific to the analysis of the ordinal outcome.

Results from this review will outline the current state of practice of the use of ordinal outcomes in RCTs. Ways to improve the analysis and reporting of ordinal outcomes in RCTs will be discussed.

**Methods and analysis:**

We will review RCTs that are published in the top four medical journals (*British Medical Journal*, *New England Journal of Medicine*, *The Lancet* and the *Journal of the American Medical Association*) between 1 January 2012 and 31 July 2022 that use an ordinal outcome as either a primary or a secondary outcome. The review will identify articles through a PubMed-specific search strategy. Our review will adhere to guidelines for scoping reviews as described in the PRISMA-ScR checklist. The study characteristics and details of the study design and analysis, including the target parameter(s) and statistical methods used to analyse the ordinal outcome, will be extracted from eligible studies. The screening, review and data extraction will be conducted using Covidence, a web-based tool for managing systematic reviews. The data will be summarised using descriptive statistics.

**Supplementary Information:**

The online version contains supplementary material available at 10.1186/s13063-023-07262-8.

## Background

Randomised controlled trials (RCTs) typically aim to estimate the average causal effect of one intervention relative to at least one other intervention. The average causal effect can be summarised using a number of target parameters, such as a risk or odds ratio (for a binary outcome) or difference in means (for a continuous outcome). The outcome of interest in a trial may be nominal, ordinal, interval or ratio [1]. Nominal outcomes are outcomes that are categorical and unranked, for example the blood type of a patient. If the outcome is measured on an interval scale, the outcome can be categorised and ranked, and the difference between any two proximate values are equally spaced (e.g. body temperature). In addition to these properties, the ratio scale has a true zero point (e.g. weight). Ordinal scales can be considered to inhabit the space between nominal and interval/ratio scales; they are categorised and ranked, but the distance between any two categories is not necessarily meaningfully quantifiable or equally spaced [2]. For example, a change from a disease-free state to hospitalisation would not necessarily be considered to be equivalent to a change from hospitalisation to death. The categories should also be mutually exclusive (such that the categories are non-overlapping), detect improvement and deterioration, and be unambiguously defined (so that categories can be clearly distinguished from each other) [3]. An additional condition of an ordinal outcome, if it measures a change between two points in time, is that the scale should be symmetrical in structure to avoid bias [3]. That is, there should be an equal number of categories that represent both improvement and deterioration.

Ordinal outcomes have become increasingly common in trials, particularly during the COVID-19 pandemic. For example, the World Health Organization (WHO) developed the WHO Clinical Progression Scale [4], an ordinal scale that describes disease severity of COVID-19 that has been adapted in various treatment trials [5, 6]. The categories reflect patient states that include being uninfected with COVID-19, ambulatory mild disease (asymptomatic or symptomatic), moderate disease (defined by patient hospitalisation and whether oxygen therapy is required or not), severe disease (ranging from a hospitalised patient who requires oxygen by non-invasive ventilation or high flow, to patients requiring mechanical ventilation with any of vasopressors, dialysis or extracorporeal membrane oxygenation for treatment), and death [4]. A review of clinical trials on the management of COVID-19 found that over half of the trials that evaluated disease severity and progression used an ordinal outcome [7], where the majority were used as secondary outcomes. Ordinal outcomes are also commonly used in stroke trials that often use the modified Rankin scale, a measure of the degree of disability among individuals who have suffered a stroke, as an outcome of interest [8–11].

Although it can be easier to interpret a clinically important effect using a dichotomised or continuous outcome, various disease states measured on an ordinal scale can represent meaningful distinctions that may be of clinical importance. Ordinal outcomes can also retain information and increase statistical power compared to dichotomised outcomes, allowing a smaller sample size to be used [12]. They can also answer important clinical questions regarding specific patient states that cannot be answered using continuous outcomes, and can allow multiple clinical outcomes to be comprised in a single endpoint. Although there are advantages to using ordinal outcomes, the required analyses can be complicated and important considerations need to be made in the design phase of the trial. Examples of such considerations could be the number of categories in the ordinal scale (fewer categories may reduce power and increase the sample size needed to detect an effect [13]), and the need to define an appropriate target parameter to compare the intervention groups.

There are a number of different target parameters that may be used to compare interventions with an ordinal outcome. For example, one could use an odds ratio that is assumed to be constant across all of the dichotomisations of the ordinal scale, known as the proportional odds assumption. Such a statistic can be estimated using the cumulative logit model, with the resulting model being commonly referred to as the ‘proportional odds model’ [14]. Alternatively, the target parameters of interest might be odds ratios that use a baseline category as the reference level that can be analysed using multinomial regression. Ordinal outcomes can also be dichotomised for analysis, with the target parameter of interest possibly being the difference in proportions between the intervention groups that can be estimated using binomial regression. Finally, the ordinal outcome can be treated as continuous data, with the enumerating index of each category serving as a continuous endpoint, with the target parameter of interest being a difference in means, estimated using a linear regression model.

There are pros and cons to the different target parameters and methods of analysis for ordinal outcomes. When an ordinal outcome has been dichotomised for analysis, the analyses are simple and the interpretation of the effect of interest can be easily understood. Ordinal outcomes that have been dichotomised, however, can discard potentially useful information on the levels of the scale and can lower statistical power compared to the original ordinal outcome [15–17]. Armstrong and Sloan found that the variance of the odds ratio using a logistic regression model is between 25–50% higher than the variance of the odds ratio estimated from a cumulative logit model [18], therefore reducing the power to detect a clinically important effect. When an ordinal outcome is treated as a continuous outcome, a linear regression model can be used to estimate a difference in means. Such an analysis assumes that the difference between any two categories of the ordinal outcome is uniform and separated by a quantifiable distance, the outcome has unbounded support, and that the outcome follows a normal distribution. Although the analyses are straightforward, these assumptions are likely to be violated as ordinal outcomes often have few categories that is insufficient to approximate a normal distribution and, more importantly, the distance between any two categories cannot be described quantitatively. Any treatment effect estimated from such an analysis would therefore be difficult to interpret.

If the target parameter is an odds ratio from a proportional odds model, then the target parameter has a fairly simple clinical interpretation (usually being the odds of a better outcome). However, in practice, the proportional odds assumption may not hold [19, 20], in which case the odds ratios across each binary split of the ordinal outcome are not equal. Instead, the treatment comparison can be extended to be odds ratios that are not constant across the binary splits of the ordinal scale, which can be estimated using a partial proportional odds model [21]. Alternatively, adjacent-category logit and continuation ratio models could also be used to estimate the odds ratios, though these models have different model assumptions and interpretations of the target parameter(s). All these models also have natural extensions to account for repeated measures over time, for example via mixed models [22] or Markov transition models [23, 24].

There has been some methodological research that describe how ordinal outcomes can be used in in specific settings, such as vascular prevention trials [25] and comparative studies [26]. However, these studies focussed on a small number of statistical models and are not reflective of more general settings. The increasing use of ordinal outcomes in randomised trials demands an improved understanding of how ordinal outcomes are used in practice. Better understanding will ensure that any issues in the use of ordinal outcomes in RCTs are identified and improvements to the reporting and analyses of such outcomes can be discussed. We aim to improve understanding by conducting a scoping review to systematically review the literature to *(i)* identify which target parameters are of interest in trials that use an ordinal outcome and whether these are explicitly defined; *(ii)* describe how ordinal outcomes are analysed in RCTs to estimate a treatment effect; and *(iii)* describe whether RCTs that use an ordinal outcome adequately report key methodological aspects specific to the analysis of the ordinal outcome.

## Methods

### Search strategy

We anticipate that the expected start date of this review is 15 August 2022 and the anticipated completion date is 1 March 2023. We will systematically search, identify and describe RCTs that have used an ordinal outcome that have been published in the top four medical journals between 1 January 2012 and 31 July 2022. The four medical journals that will be included in this search are *British Medical Journal* (BMJ), *New England Journal of Medicine* (NEJM), *The Lancet* and the *Journal of the American Medical Association* (JAMA). These journals have been selected because they are the top journals in the medical field that publish original, peer-reviewed research from RCTs and have been used in other reviews of trial methodology [27, 28]. It is expected that these journals will capture the current best practice in the use of ordinal outcomes in RCTs.

We will identify RCTs to be included in the review by searching PubMed. Our search terms will employ search strategies developed to identify RCTs [29], and terms that are used to describe ordinal outcomes in the title and abstract of relevant published articles. Since we anticipate that varied terminologies are used to describe ordinal outcomes, we first examined various RCTs that use an ordinal outcome to determine the type of terminology that is used to describe ordinal outcomes. This enabled us to develop and refine our search strategy. The full search strategy to be used in the review is outlined in Table 1.

**Table 1:** PubMed Search Strategy

Search strategy	
(JAMA[journal] OR NEJM[journal] OR lancet[journal] OR BMJ[journal]) AND (ordinal[tiab]^1^ OR categorical[tiab] OR multinomial[tiab] OR “item-response” [tiab] OR psychometric[tiab] OR scale[tiab] OR Likert[tiab]) AND (randomized controlled trial[pt]^2^ OR controlled clinical trial[pt] OR trial[tiab] OR randomized[tiab] OR placebo [tiab] OR clinical trials as topic[mesh: noexp]^3^ OR randomly[tiab])	

^1^Indicates that the search is conducted on article titles and abstracts only

^2^Corresponds to a publication type to indicate the article’s type of information conveyed

^3^Corresponds to a medical subject headings such that the explosion feature has been turned off (explosion searches the more specific terms beneath that heading)

### Eligibility criteria

#### Inclusion criteria

The review will include studies that meet the following criteria:

1. The study includes at least one RCT. We will use the Cochrane definition of an RCT, which are studies in which one of two (or more) health interventions are prospectively assigned to individuals using a random/quasi-random method of allocation [29].
2. The study was published in one of the top four medical journals between 1 January 2012 and 31 July 2022: *British Medical Journal*, *New England Journal of Medical*, *Journal of the American Medical Association* or *The Lancet*. For articles with more than one publication date (such as early-view/online publication), only one publication date is required to be between 1 January 2012 and 31 July 2022. If two or more publication dates are between these dates, the earlier date will be recorded.
3. The study reports an analysis of a primary or secondary ordinal outcome. Our review will focus on any ordinal outcomes that are used, whether they were specified as a primary or secondary outcome. The target ordinal outcome must have multiple, monotonically ordered categories that are not necessarily separated by a quantifiable distance and do not have equal spacing between categories.

#### Exclusion criteria

We will exclude studies that meet the following criteria:

1. The study is written in a language other than English. This criteria has been included as we are not capable of translating studies written in other languages.
2. The study is a methodological paper examining data from an RCT. This criterion is included because we are only interested in how ordinal outcomes have been used in real-world RCTs. Methodological papers tend to provide motivating examples that may not be representative of RCTs that use an ordinal outcome in practice.
3. The study does not provide either an abstract or full-text.
4. The study analyses data from non-human subjects only.
5. The manuscript provides a commentary, review, opinion or description only.
6. The manuscript is a protocol or statistical analysis plan. These manuscripts will be excluded from the review since one of the aims of this review is about what statistical models were reported in the analysis, and whether they have checked and justified the model assumptions.
7. The only ordinal outcome(s) is(are) measured on an interval scale. Studies will be excluded if the ordinal outcome is a numeric scale in which differences between proximate values are separated by a quantifiable and equal distance (e.g. the visual analogue scale). Similarly, studies will be excluded if the outcomes were derived from multiple items measured on an ordinal scale in which the summary variable is also interval data, such as the Hamilton Depression Rating Scale. Outcomes that are inherently interval data can be analysed using conventional and valid statistical methods, such as linear regression. The focus of this review is how ordinal outcomes, whose categories are not equally spaced and any meaningful distance between categories can only be described qualitatively, can be appropriately analysed in RCTs.
8. The study is a systematic review and/or provides a meta-analyses of RCTs.

### Sample size

There is no pre-defined sample size. We plan to include all eligible studies that appear in our search using our pre-defined search strategy.

### Study selection

Titles and abstracts identified by the search strategy will be extracted into Covidence, a web-based tool for managing systematic reviews [30]. The review began with a piloting phase, where two authors (CS, RM) independently assessed 20 abstracts to ensure that the application of the inclusion and exclusion criteria was consistent between reviewers. If there was more than minor disagreement, then the criteria were further refined following discussion between the three reviewers (CS, RM, KL).

The review will be conducted by two authors (CS and one of RM or KL, or a substitute reviewer) through a two-phase screening process. In the first phase, all abstracts will be screened by one author (CS). A 10% random sample of the identified abstracts will be screened by a second author (either RM or KL) to identify those for inclusion. If there is disagreement over whether a study should be included between reviewers, then the study will move to the second phase of screening where the full text will be evaluated against the eligibility criteria by both reviewers and inclusion will be determined via discussion among the 3 reviewers. Studies that are found to have met all the inclusion and none of the exclusion criteria will be included.

### Data extraction and management

Covidence will be used to extract and store the data from the review. A data extraction questionnaire was developed (additional file 1) and was piloted for use by CS and RM using a sample of 10 studies, with changes made to the questionnaire where necessary. CS will extract data from all eligible studies in the review. Double data extraction will be performed by either RM or KL on a random sample of 10% of eligible studies and additionally when there is uncertainty about studies to ensure consistency throughout the data extraction process.

A full list of the data extraction items is provided in Table 2. We will only extract data that is reported in the article and additional material. We expect some data will be challenging to extract. The assumptions and simplifications that we will make under these conditions are summarised in Additional file 2. If any post hoc assumptions or simplifications are made, these will be reported as part of the analysis.

**Table 2:** Summary of items reported in an article that will be extracted

Category	Extracted data
**Study characteristics**	• Title
	• First author name(s)
	• Publication year
	• Funding source
	• Journal
	• Trial type
**Subject matter**	• Setting
	• Medical condition studied
	• Medical specialty studied
**Design**	• Ordinal outcome type
	• Number of categories in the outcome
	• Whether the ordinal outcome was measured at a single time point or as a measure of change
	• Whether the categories of the ordinal scale were clearly defined, ordered, mutually exclusive and, if a measure of change, symmetrical
	• Whether the ordinal outcome was a primary or secondary outcome
	• Whether sample size determination was used based off the ordinal outcome
	• Number of study participants included in the analysis (largest if multiple analyses)
**Statistical methods**	• The statistical model(s) or method(s) that were used to analyse the ordinal outcome
	• Type of inference used (frequentist/Bayesian)
	• Target parameter used
	• How the distribution of the ordinal outcome was summarised by intervention
	• Methods used to account for repeated measures over time (if applicable)
	• Details on whether the model assumptions were reported
	• Whether the analysis that was reported in the results differed from the analysis
	outlined in the methods section of the manuscript
**Software included**	• Statistical software package used for the analysis

### Analysis

Once data extraction is complete, the data will be exported from Covidence. The extracted data will be cleaned and analysed in Stata version 17.0 [31]. The data will be summarised using descriptive statistics. Frequencies and percentages will be reported to summarise categorical data. Medians and interquartile ranges will be used to summarise continuous data. We will synthesise qualitative data in a narrative format. The data and code will be made available publicly on Github, an online software repository.

### Patient and public involvement

There will be no patients or public involvement in this review.

### Reporting

The findings from this review will be reported using the Preferred Reporting Items for Systematic Reviews and Meta-Analyses extension for Scoping Reviews (PRISMA-ScR) checklist [32].

## Discussion

This paper describes a protocol for a scoping review that aims to examine the use, analysis and reporting of ordinal outcomes in RCTs in the top four medical journals. To our knowledge, there has not been a review of how ordinal outcomes are used in RCTs, particularly in the last decade. This review aims to address this gap and identify how ordinal outcomes are used in trials to improve our understanding of the appropriate analysis of such outcomes.

### Strengths and limitations

A targeted review of RCTs in top medical journals publishing original and recent research will highlight the current state of practice for analysing ordinal outcomes. We have a priori specified eligibility criteria and strategies to handle anticipated challenges with data extraction. The screening and data extraction process will be conducted systematically, in which the pilot tests and double data extraction ensure the consistency and reliability of the extracted data. The search strategy, dataset and code will be made publicly available to ensure that the review is reproducible. The PRISMA-ScR checklist will be used to ensure that reporting is conducted to the highest standard.

This review will have its limitations. Although there is an exclusive focus on the PubMed database and on the top four medical journals, we made this decision given the scoping nature of the review, to make it as reproducible as possible, and to ensure that the total number of studies included in the review was manageable. The results reported within these journals are likely to inform clinical practice and therefore are likely to reflect how research using ordinal outcomes is conducted with a primarily clinical aim. Our search strategy may miss certain phrases or variants (particularly related to an ordinal outcome), although our piloting phase has hopefully mitigated this to a large degree. We deliberately avoided including the names of specific scales in our search strategy as this would provide a non-representative sample.

### Implications of this research

In addition to critically appraising and examining the literature regarding the use of ordinal outcomes in RCTs, this review will identify areas of improvement for the use and the analysis of ordinal outcomes for future trials to ensure the reliability and transparency of reporting of such outcomes. We also hope the results will be used to inform methodological research in the analysis of ordinal outcomes.

## Supplementary information

**Additional file 1.** Data extraction questionnaire. This is a copy of the data extraction questionnaire that will be used for this review in PDF format.

**Additional file 2.** Anticipated challenges with data extraction and how they will be handled. This is a table that outlines that anticipated challenges with data extraction in PDF format.

## Data Availability

Not applicable (no data collected). This protocol is available as a pre-print on ArXiv [33].

## Abbreviation

RCT: Randomised controlled trial

## References

[1] Stevens SS (1946). On the theory of scales of measurement. Science..

[2] Velleman PF, Wilkinson L (1993). Nominal, ordinal, interval, and ratio typologies are misleading. Am Stat..

[3] MacKenzie CR, Charlson ME (1986). Standards for the use of ordinal scales in clinical trials. Br Med J (Clin Res Ed)..

[4] Marshall JC, Murthy S, Diaz J, Adhikari N, Angus DC, Arabi YM (2020). A minimal common outcome measure set for COVID-19 clinical research. Lancet Infect Dis..

[5] Lovre D, Bateman K, Sherman M, Fonseca VA, Lefante J, Mauvais-Jarvis F (2021). Acute estradiol and progesterone therapy in hospitalised adults to reduce COVID-19 severity: a randomised control trial. BMJ Open..

[6] Song AT, Rocha V, Mendrone-Júnior A, Calado RT, De Santis GC, Benites BD (2022). Treatment of severe COVID-19 patients with either low-or high-volume of convalescent plasma versus standard of care: A multicenter Bayesian randomized open-label clinical trial (COOP-COVID-19-MCTI). Lancet Reg Health Am..

[7] Mathioudakis AG, Fally M, Hashad R, Kouta A, Hadi AS, Knight SB (2020). Outcomes evaluated in controlled clinical trials on the management of COVID-19: a methodological systematic review. Life..

[8] Banks JL, Marotta CA (2007). Outcomes validity and reliability of the modified Rankin scale: implications for stroke clinical trials: a literature review and synthesis. Stroke..

[9] de la Ossa NP, Abilleira S, Jovin TG, García-Tornel Á, Jimenez X, Urra X (2022). Effect of Direct Transportation to Thrombectomy-Capable Center vs Local Stroke Center on Neurological Outcomes in Patients With Suspected Large-Vessel Occlusion Stroke in Nonurban Areas: The RACECAT Randomized Clinical Trial. JAMA..

[10] Hubert GJ, Hubert ND, Maegerlein C, Kraus F, Wiestler H, Müller-Barna P (2022). Association between use of a flying intervention team vs patient interhospital transfer and time to endovascular thrombectomy among patients with acute ischemic stroke in nonurban Germany. JAMA..

[11] Bösel J, Niesen WD, Salih F, Morris NA, Ragland JT, Gough B (2022). Effect of early vs standard approach to tracheostomy on functional outcome at 6 months among patients with severe stroke receiving mechanical ventilation: the SETPOINT2 Randomized Clinical Trial. JAMA..

[12] Roozenbeek B, Lingsma HF, Perel P, Edwards P, Roberts I, Murray GD (2011). The added value of ordinal analysis in clinical trials: an example in traumatic brain injury. Crit Care..

[13] Peterson RL, Vock DM, Powers JH, Emery S, Cruz EF, Hunsberger S (2017). Analysis of an ordinal endpoint for use in evaluating treatments for severe influenza requiring hospitalization. Clin Trials..

[14] McCullagh P (1980). Regression models for ordinal data. J R Stat Soc Ser B Methodol..

[15] Altman DG, Royston P (2006). The cost of dichotomising continuous variables. BMJ..

[16] D’Amico G, Abraldes JG, Rebora P, Valsecchi MG, Garcia-Tsao G (2020). Ordinal outcomes are superior to binary outcomes for designing and evaluating clinical trials in compensated cirrhosis. Hepatology..

[17] Dodd LE, Follmann D, Wang J, Koenig F, Korn LL, Schoergenhofer C (2020). Endpoints for randomized controlled clinical trials for COVID-19 treatments. Clin Trials..

[18] Armstrong BG, Sloan M (1989). Ordinal regression models for epidemiologic data. Am J Epidemiol..

[19] Kim JH (2003). Assessing practical significance of the proportional odds assumption. Stat Probab Lett..

[20] Fullerton AS, Xu J (2012). The proportional odds with partial proportionality constraints model for ordinal response variables. Soc Sci Res..

[21] Peterson B, Harrell FE (1990). Partial proportional odds models for ordinal response variables. J R Stat Soc Ser C Appl Stat..

[22] Agresti A, Lang JB (1993). A proportional odds model with subject-specific effects for repeated ordered categorical responses. Biometrika..

[23] de Lara I, Hinde J, De Castro A, Da Silva I (2017). A proportional odds transition model for ordinal responses with an application to pig behaviour. J Appl Stat..

[24] Mhoon KB, Chan W, Del Junco DJ, Vernon SW (2010). A continuous-time Markov chain approach analyzing the stages of change construct from a health promotion intervention. JP J Biostat..

[25] Bath PM, Geeganage C, Gray LJ, Collier T, Pocock S (2008). Use of ordinal outcomes in vascular prevention trials: comparison with binary outcomes in published trials. Stroke..

[26] Scott SC, Goldberg MS, Mayo NE (1997). Statistical assessment of ordinal outcomes in comparative studies. J Clin Epidemiol..

[27] Bell ML, Fiero M, Horton NJ, Hsu CH. Handling missing data in RCTs; a review of the top medical journals. BMC Med Res Methodol. 2014;14(1):1–8. 10.1186/1471-2288-14-118 PMC4247714 25407057

[28] Berwanger O, Ribeiro RA, Finkelsztejn A, Watanabe M, Suzumura EA, Duncan BB (2009). The quality of reporting of trial abstracts is suboptimal: survey of major general medical journals. J Clin Epidemiol..

[29] Higgins JP, Thomas J, Chandler J, Cumpston M, Li T, Page MJ, et al. Cochrane handbook for systematic reviews of interventions. Wiley. 2019. 10.1002/14651858.ED000142 PMC10284251 31643080

[30] Veritas Health Innovation. Covidence systematic review software. Melbourne: Australia; 2020.

[31] StataCorp L. Stata statistical software: Release 17 (2021). College Station: StataCorp LP; 2021.

[32] Tricco AC, Lillie E, Zarin W, O’Brien KK, Colquhoun H, Levac D (2018). PRISMA extension for scoping reviews (PRISMA-ScR): checklist and explanation. Ann Intern Med..

[33] Selman CJ, Lee KJ, Mahar, RK. Statistical analyses of ordinal outcomes in randomised controlled trials: protocol for a scoping review. arXiv preprint arXiv:2208.06154. 2022. 10.1186/s13063-023-07262-8 PMC10119829 37085929

