# Supplementary material for: Statistical analyses of ordinal outcomes in randomised controlled trials: protocol for a scoping review
Source: Trials. 2023 Apr 21;24:286. doi: 10.1186/s13063-023-07262-8 (PMC10119829; doi:10.1186/s13063-023-07262-8)
Supplement: Supplementary file 1 — Additional file 1. Data extraction questionnaire. This is a copy of the data extraction questionnaire that will be used for this review in PDF format. [file 13063_2023_7262_MOESM1_ESM.pdf]

5 Supplementary Material

Supplementary Material 1: Data Extraction Questionnaire

Study Characteristics

1. What is the title of the manuscript?

2. What is the name of the first author?

3. What was the year of publication?

(1) 2012
(2) 2013
(3) 2014
(4) 2015
(5) 2016
(6) 2017
(7) 2018
(8) 2019
(9) 2020
(10) 2021
(11) 2022

4. a) What was the funding source? (tick all that apply)

(1) Non-profit
(2) Not reported
(3) Public
(4) Industry
(9) Other
(99) Unclear/unknown

4. b) If other, specify the other funding source.

5. What journal was the article published?

(1) BMJ
(2) JAMA
(3) The Lancet
(4) NEJM

6. Was an adaptive design used in the trial?

(0) No

(1) Yes
(99) Unknown/could not be determined

Subject Matter

1. Was the study conducted in a clinical setting?

(1) Clinical setting
(2) Non-clinical setting

2. If the study was conducted in a clinical setting, what medical condition was under study?

3. If the study was conducted in a clinical setting, what was the medical specialty/field?

Design

1. What type of ordinal scale was used?

(1) Single-state ordinal scale
(2) Transition-state ordinal scale
(99) Unknown/could not be determined

2. Was the ordinal outcome a primary or secondary outcome?

(1) Primary outcome
(2) Secondary outcome

3. How many categories did the ordinal outcome have prior to the analysis (and prior to dichotomisation, if appliocable)? (record as an integer, N/A if unclear)

4. What properties of an ordinal scale did the ordinal outcome satisfy? (tick all that apply)

(1) Clearly defined and unambiguous categories
(2) Mutually exclusive categories
(3) Categories ordered in a hierarchical manner
(4) The ordinal scale used can detect improvement and deterioration
(5) Symmetrical scale (if the ordinal outcome is a transition-state scale)
(99) Unknown/could not be determined

5. a) What did the ordinal outcome measure?

(1) Mortality/survival
(2) Clinical outcomes (e.g. (time to) treatment success/failure, severity scores, symptoms)
(3) Physiological outcomes (e.g. viral detection/load, biomarkers)
(4) Adverse events

(5) Life impact (e.g. quality of life, compliance, mental health, satisfaction)
(6) Resource use (e.g. economic, hospital, need for further intervention)
(9) Other
(99) Unknown/could not be determined

5. b) If other, describe what the ordinal outcome measured.

6. a) Was the sample size determined in advance based on the original ordinal scale? (if the outcome was dichotomised for the analysis and the sample size was determined based off the binary outcome, select 'No')

(0) No
(1) Yes
(99) Unknown/could not be determined

6. b) If yes, what methods did the authors use to determine the sample size?

(1) Analytical
(2) Simulation
(3) Ad-hoc
(9) Other
(99) Not applicable

7. What was the number of study participants included in the model used to analyse the ordinal outcome (largest N if multiple analyses)? (record as an integer)

Statistical methods

1. a) How was the distribution of the ordinal outcome summarised by intervention group? (tick all that apply)

(1) Odds (category-specific)
(2) Frequencies and/or proportions/percentages (category-specific; tick yes even if the categories were dichotomised)
(3) Means (across all categories)
(4) Medians (across all categories)
(5) Standard deviations (across all categories)
(6) Interquartile ranges (across all categories)
(7) Summaries by group not used
(9) Other
(999) Unknown/could not be determined

1. b) If other, describe how the distribution of the ordinal measure was summarised by intervention group.

2. If inferential statistics were used to analyse the ordinal outcome, what type of inference was used?

(1) Frequentist inference
(2) Bayesian inference
(99) Unknown/could not be determined
(999) Not applicable

3. a) What was the reported target parameter? (tick all that apply)

(1) Odds ratio(s)
(2) Difference in means
(3) Difference in medians
(4) Risk difference
(5) Risk ratio
(6) Descriptive analysis only
(7) Non-parametric procedure used
(9) Other
(99) Unknown/could not be determined

3. b) If other, specify the reported target parameter.

4. a) Which statistical model(s) or method(s) did the authors report to analyse the ordinal outcome? (tick all that apply)

(1) Cumulative logit model
(2) Continuation ratio model
(3) Ordinal probit model
(4) Adjacent category logit model
(5) Logistic model
(6) Linear model
(7) Baseline category logit model
(8) Cox proportional hazards model
(9) Mann-Whitney U-test
(10) Cochran-Mantel-Haenszel test
(99) Other
(999) None
(9999) Unknown/could not be determined

4. b) If other, specify the statistical model or method that was used to analyse the ordinal outcome.

5. a) Did the authors report in the manuscript whether a different statistical model or method had to be used in the analysis of the ordinal outcome? (e.g. the authors used a partial proportional odds model due to violation of the proportional odds assumption)

(0) No
(1) Yes
(99) Unknown
(999) Not applicable

5. b) If yes, provide detail as to which statistical model(s) or method(s) were in the initial analysis of the ordinal outcome and explain, if possible, why these were not used by the authors in the reported analysis.

6. a) Was the validity of the assumptions required for the reported statistical model/method(s) checked, justified and clearly described by the authors?

(0) No
(1) Yes
(99) Unknown/could not be determined
(999) Not applicable

6. b) If yes, how did the authors check the assumptions of the statistical model/method? (tick all that apply)

(1) Graphical methods
(2) Statistical methods
(3) Prediction methods
(9) Other
(99) Unknown/could not be determined
(999) Not applicable

6. c) If other, please describe.

7. a) If the ordinal outcome was measured repeatedly over time, how did the authors account for repeated measures in the analysis of the ordinal outcome? (tick all that apply)

(1) Robust standard errors
(2) Generalised estimating equations
(3) Mixed effects models
(4) Discrete-time Markov transition models
(5) Continuous-time Markov transition models
(6) Adjusted for baseline measurement
(7) No methods used to account for repeated measures
(9) Other
(99) Unknown/could not be determined
(999) Not applicable (i.e. only one measure was used in the analysis of the outcome)

7. b) If other, provide details.

Software

1. What statistical software package was used for the analysis? (tick all that apply)

(1) R

(2) SAS
(3) SPSS
(4) Stata
(9) Other
(99) Unknown/not stated

1. b) If other, specify what software was used.

Other

1. Detail any other information that is deemed relevant and useful for the review.
