# Supplementary material for: Statistical analyses of ordinal outcomes in randomised controlled trials: protocol for a scoping review
Source: Trials. 2023 Apr 21;24:286. doi: 10.1186/s13063-023-07262-8 (PMC10119829; doi:10.1186/s13063-023-07262-8)
Supplement: Supplementary file 2 — Additional file 2. Anticipated challenges with data extraction and how they will be handled. This is a table that outlines that anticipated challenges with data extraction in PDF format. [file 13063_2023_7262_MOESM2_ESM.pdf]

Supplementary Table 1: Anticipated challenges with data extraction and how they will be handled.

**Challenge for data extraction**	**Category of items affected**	**How challenge will be handled**
Articles may have more than one publication.	Inclusion criteria	For articles with more than one publication date (such as early-view/online publication or preliminary results) only one publication date is required to be between 1 January 2012 and 31 July 2022. The earlier date will be recorded if two or more publication dates are between 1 January 2012 and 31 July 2022. If there are two publications that include a preliminary reporting of results followed by the main reporting of results, then data from the main article will be extracted.
There are multiple trials in the same manuscript.	Inclusion criteria	If there are multiple trials included in the same manuscript but are all sufficiently different from each other, all trials that use an ordinal outcome will be included in the review. If any trials are very similar to each other (e.g. share the same protocol and/or statistical analysis plan), the first trial that is mentioned and uses an ordinal outcome will be included in the review. If the results of multiple trials are pooled, then data will be extracted as if it were coming from a single trial.
If a study uses more than one statistical model to analyse the ordinal outcome.	Data extraction (statistical methods)	All statistical models/methods will be recorded. For example, it is possible that a study initially describes in the methods section that a proportional odds model will be used. However, if the proportional odds assumption was violated, the study may use an alternative model to analyse the outcome in the final analysis. We will only report statistical methods that were reported in the final analysis.
There are multiple ordinal outcomes that are used in the study.	Any data extraction categories	If there are multiple ordinal outcomes, it is likely that the study will use similar, if not the same, statistical methods to analyse the outcome. Consequently, we will only examine how the study has used the first ordinal outcome for the data extraction process.
There are two or more target parameters used for the same ordinal scale.	Any data extraction categories	It is reasonable to assume that a study would be interested in potentially more than one target parameter using the same ordinal scale if, for example, the study was investigating both the proportion of absolute attainment of a pre-specified outcome, and if there was an improvement in the distribution of scores on the original ordinal scale. If an article has included two or more target parameters using the same ordinal scale, we will extract data only for the outcome that is analysed on the original ordinal scale and/or the results are summarised together (e.g. we will not extract data if the primary outcome is a dichotomised version of the ordinal outcome and the secondary outcome analyses the ordinal scale).
